# High Genetic Diversity and Low Differentiation of *Michelia coriacea* (Magnoliaceae), a Critically Endangered Endemic in Southeast Yunnan, China

**DOI:** 10.3390/ijms13044396

**Published:** 2012-04-10

**Authors:** Xingfeng Zhao, Yongpeng Ma, Weibang Sun, Xiangying Wen, Richard Milne

**Affiliations:** 1 Kunming Botanical Garden, Kunming Institute of Botany, Chinese Academy of Sciences, Kunming 650204, China; E-Mails: xingfengzhao@ffichina.org (X.Z.); mayongpeng@mail.kib.ac.cn (Y.M.); 2 Fauna & Flora International China Programme, Beijing 100101, China; 3 South China Botanical Garden, Chinese Academy of Sciences, Guangzhou 510650, China; E-Mail: xiangyingwen@mail.scib.ac.cn; 4 Institute of Molecular Plant Sciences, University of Edinburgh, Edinburgh EH9 3JH, UK; E-Mail: r.milne@ed.ac.uk

**Keywords:** *Michelia coriacea*, genetic diversity, critically endangered plant, ISSR markers, SSR markers

## Abstract

*Michelia coriacea*, a critically endangered tree, has a restricted and fragmented distribution in Southeast Yunnan Province, China. The genetic diversity, genetic structure and gene flow in the three extant populations of this species were detected by 10 inter-simple sequence repeat (ISSR) markers and 11 simple sequence repeat (SSR) markers. Examination of genetic diversity revealed that the species maintained a relatively high level of genetic diversity at the species level (percentage of polymorphic bands) PPB = 96.36% from ISSRs; PPL (percentage of polymorphic loci) = 95.56% from SSRs, despite several fragmental populations. Low levels of genetic differentiation among the populations of *M. coriacea* were detected by Nei’s *G*_st_ = 0.187 for ISSR and Wright’s *F*_st_ = 0.090 for SSR markers, which is further confirmed by Bayesian model-based STRUCTURE and PCoA analysis that could not reveal a clear separation between populations, although YKP was differentiated to other two populations by ISSR markers. Meanwhile, AMOVA analysis also indicated that 22.84% and 13.90% of genetic variation existed among populations for ISSRs and SSRs, respectively. The high level of genetic diversity, low genetic differentiation, and the population, structure imply that the fragmented habitat and the isolated population of *M. coriacea* may be due to recent over-exploitation. Conservation and management of *M. coriacea* should concentrate on maintaining the high level of genetic variability through both *in* and *ex-situ* conservation actions.

## 1. Introduction

The ultimate goal of conservation biology is to maintain the evolutionary potential of species by maintaining natural levels of genetic diversity [1–4]. To achieve this goal, understanding of the species’ genetic diversity and genetic structure is necessary [5]. Within a species, genetic diversity of plant populations is largely determined by factors such as mating system, gene flow, genetic drift, evolutionary and life history [6–8]. Compared with widespread taxa, many rare and endangered species may become genetically depressed because of their small population size [9]. Habitat fragmentation exacerbates these problems, and has therefore been recognized as one of the greatest threats to the survival of many species in small and/or isolated populations [10]. Knowledge of genetic diversity and population age structure in rare plants not only enhances our understanding of population dynamics, adaptation and evolution, but also provides useful information for biological conservation [11,12].

The recently described *Michelia coriacea* (Magnoliaceae) is a critically endangered species, restricted to karst areas of Southeast Yunnan Province, China, bordering northern Vietnam [13–15]. This area has exceptionally high species diversity, and has been recognized as one of the most important biodiversity hotspots in China [16,17], but has been severely affected by habitat fragmentation due to human activities, causing populations of rare plants to become isolated from each other.

**Figure 1:**
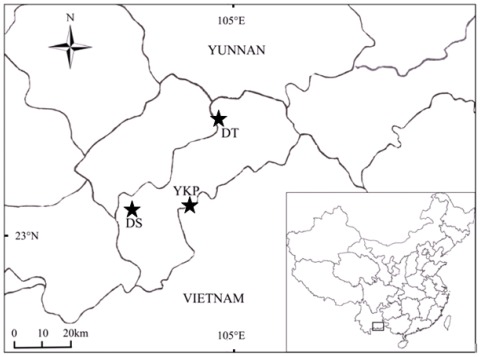
Map of the three extant *Michelia coriacea* populations in Southeast Yunnan, China. Populations are DS: Daping-Shipen; YKP: Yang-Kai-Ping; and DT: Dongma-Tiechang.

Currently, *M. coriacea* occurs as scattered individuals in evergreen woods on limestone slopes or roadsides at altitude of 1300–1600 m, and has a potential distribution range of approximate 4190 km^2^ [18]. These individuals are distributed across only three extant populations: Daping and Shipen, Dongma and Tiechang, and Yang-Kai-Ping (defined as populations of DS, DT and YKP in this paper) (Figure 1). In DS population, some individuals of *M. coriacea* are over 100 years old, having been protected as symbols of “good fortune” by local villagers; however, they are surrounded and isolated by farmland and villages. In DT population, the maximum age of sampled trees was less than 30 years, and in YKP population the average tree age was 10–50 years [19]. All individuals in these populations are in habitats that have been degraded and fragmented due to heavy logging and vegetation destruction. Undoubtedly, an understanding of the genetic diversity of the critically endangered *M. coriacea* is urgently needed for planning its conservation action.

The use of molecular analysis as an integral component in the conservation of rare and endangered species is becoming more widely used [5,11,20]. The molecular markers best suited for detecting genetic diversity should be relatively easy and inexpensive to detect and these markers should have evolved rapidly enough to be variable within populations. Inter-simple sequence repeats (ISSRs) and simple sequence repeats (SSRs) have been successfully employed and recognized as useful molecular markers in the analysis of a population’s genetic variation, and for other purposes in various species [21,22]. In order to achieve valuable and comprehensive information on the genetic diversity and population differentiation of *M. coriacea*, we employed ISSR and SSR markers to address the key issue: what are the levels of genetic diversity and differentiation within, and among, populations of *M. coriacea*? In addition, implications for conserving this critically endangered species are discussed.

## 2. Results

### 2.1. Genetic Diversity and Genetic Structure Investigated by ISSR Markers

A total of 110 bands were presented from the 10 selected primers among 58 individuals of the three populations. Of these, 106 (96.36%) were polymorphic (Table 1). At the population level, the percentage of polymorphic bands (PPB) within each population ranged from 60.00% (DS) to 81.82% (DT) with an average of 72.73% (Table 2). Populations YKP, DT and DS contained nine, eight and zero private bands respectively. Assuming Hardy-Weinberg equilibrium, the mean Nei’s gene diversity (*H*) was estimated to be 0.226 within populations and 0.283 at the species level, and the average Shannon information index (*H*_pop_) within populations and species levels was 0.345 and 0.436, respectively (Table 2).

**Figure 2:**
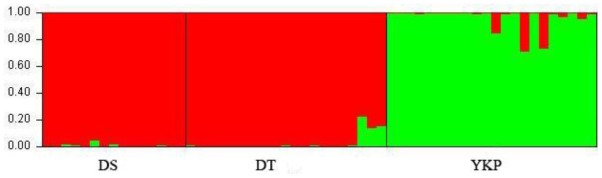
Bayesian model-based clustering STRUCTURE analysis as inferred at *K* = 2 based on ISSR data. The genotype of each individual accession is represented by a vertical line divided into colored segments, the lengths of which indicate the proportions of the genome attributed to the inferred clusters.

In the STRUCTURE analysis of ISSR data, the value of Δ*K* was 903.1 for *K* = 2, 412.9 and 233.5 for *K* = 3 and *K* = 4, respectively, and <155 for all values of *K* higher than 4. Therefore *K* = 2 best represents the data. Structure analysis run with *K* = 2 showed a clear separation between YKP and the other two populations (Figure 2).

**Figure 3:**
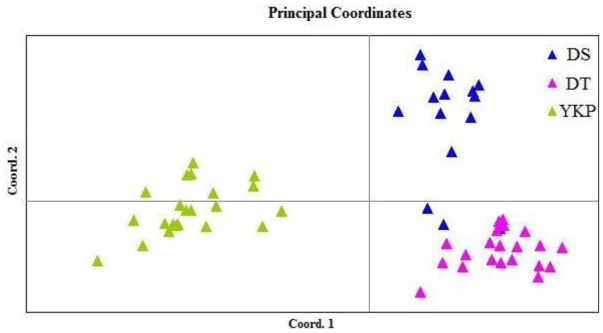
A two-dimensional plot of the Principal Coordinate Analysis (PCoA) of ISSR data showing the clustering of populations of *M. coriacea*. The first and second principal coordinates account for 39.59% and 18.88% of total variation respectively.

The two-dimensional PCoA plot (Figure 3) shows that the first principal coordinate accounts for 39.59% of total variation and separates the YKP population from the DS and DT populations. The second principal coordinate (18.88% of total variation) separated most individuals from DS from those of DT, but there was some overlap, with certain individuals from DS grouping with those from DT. AMOVA analysis among populations showed that 77.16% variation occurred within populations (Table 3). Deduced from the *G*_st_ value, the level of gene flow (*N*_m_) was estimated at 1.086 (*N*_m_ > 1), indicating no differentiation among populations.

### 2.2. Genetic Diversity and Genetic Divergence Based on SSR Markers

A total of 45 alleles at 11 SSR loci were revealed across 58 individuals of *M. coriacea*. The number of alleles per locus ranged from 2 at locus MC64 to 7 at locus MC49, with an average of 4.091 per locus (Table 4). At species level, the percentage of polymorphic loci was 95%. Values for observed heterozygosity (*H*_o_) ranged from 0.172 at locus MC45 to 0.759 locus MC8 (average 0.412), and for expected heterozygosity (*H*_e_) from 0.247 at locus MC45 to 0.782 at locus MC8 (average 0.505). PIC values ranged from 0.417 to 0.750, with an average value of 0.502 per locus (Table 4).

At the population level, the percentage of polymorphic loci per population ranged from 72.73% (DS) to 91.90% (DT) with an average of 82.15%. The average value for *H*_o_ was 0.425 (ranging from 0.335 to 0.533) and that for *H*_e_ was 0.470 (ranging from 0.429 to 0.498). Unlike private bands detected from ISSRs, private SSR alleles were present in all three populations: DS, DT and YKP contained 2, 3, and 2 private alleles respectively (Table 5). The estimated genetic differentiation among populations (*F*_st_) was 0.090 and the estimated level of gene flow (*N*_m_) among populations was 2.543. AMOVA analysis showed that 13.90% of the variance existed among populations (Table 3).

**Figure 4:**
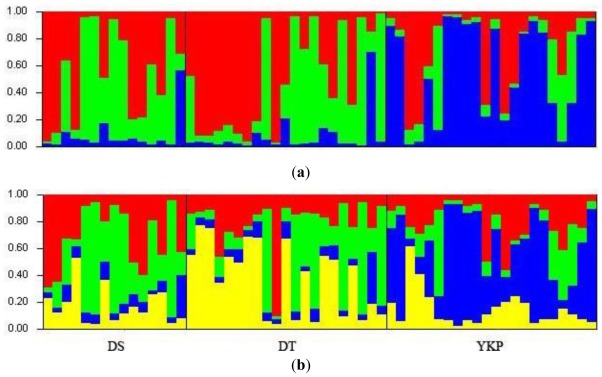
Bayesian model-based clustering STRUCTURE analysis as inferred at *K* = 3 (**a**) and *K* = 4 (**b**) based on SSR data. The genotype of each individual accession is represented by a vertical line divided into colored segments, the lengths of which indicate the proportions of the genome attributed to the inferred clusters.

In the STRUCTURE analysis of SSR data, the value of Δ*K* was 61.2 for *K* = 3, 59.3 for *K* = 4, and <51 for all values of other *K*. The scores for *K* = 3 and *K* = 4 were very similar; therefore we performed runs with each *K* value. However, results either from *K* = 3 or *K* = 4 failed to differentiate between populations (Figure 4a,b).

**Figure 5:**
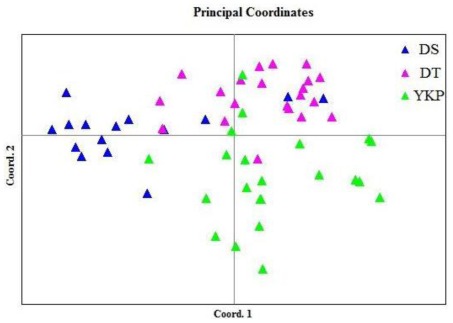
A two-dimensional plot of the Principal Coordinate Analysis (PCoA) of SSR data showing the clustering of populations of *M. coriacea*.

Similarly, PCoA analysis did not clearly separate the populations. Individuals from DS tended to score lower for coordinate 1 (accounting for 25.57% of total variation), whereas individuals from DT and YKP tended to have higher and lower scores respectively for coordinate 2 (accounting for 19.84% of total variation). However, all three populations overlapped for both coordinates, meaning that discrimination based on this data and PCoA analysis of this data was not possible (Figure 5).

Within each population, certain individuals appeared highly distinct from certain others, according to the STRUCTURE analysis of SSR data. In population DS, individuals DS1 and DS2 were highly distinct from DS5, DS6, DS8, and DS14; in DT population, DT2, DT3, DT4, DT5, DT6, DT7 and DT10 were most distinct from DT9, DT12, DT14, DT17, DT19 and DT21; in YKP population YKP1, YKP2, YKP7, YKP8, YKP9, YKP10, YKP12, YKP15, YKP16, YKP17, YKP21 and YKP22 were most distinct from YKP3, YKP4, and YKP6 (Figure 4a).

## 3. Discussion

### 3.1. Genetic Diversity in M. coriacea

The known distribution of *M. coriacea* is very narrow and fragmented, but it retains high levels of genetic diversity, mostly at the within-population level. The genetic diversity in the species overall (PPB = 96.36%, *H* = 0.283, for ISSR markers; PPL = 95.56%, *H*_e_ = 0.505, and *H*_o_ = 0.412 for SSR markers) is higher than that of its relatives *Manglietia deciduas* (PPB = 17.28%, *H* = 0.0637; [23]) and *Magnolia sieboldii* (*H*_e_ = 0.366, *H*_o_ = 0.366; [24]). In general, species with small geographic ranges tend to maintain less genetic diversity than geographically widespread species. However, exceptions are not uncommon [11,20,25,26]. Genetic diversity within populations is influenced by historical factors (e.g., founder effects, bottlenecks, extended time periods with low numbers of individuals and gene flow rates), and thus present-day population sizes may not be a reliable indication of genetic diversity.

High genetic diversity can be maintained in rare plants that are adapted to an existence comprising small isolated populations (*i.e.*, are naturally rare; [26]). However, high genetic diversity within very small populations can also be observed if very recent population size reduction has occurred, especially where that reduction has occurred within a generation or two for the species concerned; in such cases the surviving individuals are effectively samples from the larger population that existed before [11,20,27,28]. In such cases, unlike naturally rare plants, a dramatic loss of diversity in future generations is to be expected via genetic drift.

Habitat destruction appears to be recent for *M. coriacea,* as evidenced both by witness accounts of local people and the literature record. The species was first discovered in 1986 and then described as a new species in 1987 [29]. Initially, it was known from four localities in SE Yunnan, *i.e.*, DS, DT, YKP and a fourth, Guangnan [29]. Despite repeated searches of the Guangnan area between 2004 and 2006, we could not find any plants of *M. coriacea*, indicating its extinction from Guangnan within the last 30 years. However, there is no evidence for range contraction during the same period for the three extant populations of *M. coriacea* in this study. In addition, our molecular data clearly indicated apparently high levels of gene flow for *M. coriacea* (*N*_m_ = 1.086 for ISSR data, and 2.543 for SSRs), which would account for low differentiation between populations (*F*_st_ = 0.090 for SSRs and *G*_st_ = 0.187 for ISSRs). It should be noted that indirect estimates of Nm values must be interpreted with caution [30,31], and this data therefore should be viewed as general indicators of the magnitude of genetic exchange.

Low genetic differentiation and high gene flow between populations can result from long-distance gene dispersal either by pollen or by seed [10]. Long range seed dispersal has been implicated in maintaining links between populations in the rare *Michelia formosana* [32]. *M. coriacea* also has animal-dispersed seeds, and insect pollination may also have contributed to gene flow over distance in this species. However, seed or pollen flow appears unlikely between the modern, isolated populations. Therefore, gene flow between plants at the three extant sites via seeds and/or pollen was probably extensive and relatively unhindered in the past, before the populations became isolated.

### 3.2. Variation Between Populations

Populations DT (PPB = 81.82%, PPL = 91.90%) and YKP (PPB = 76.36%, PPL = 81.82%) contained relatively higher genetic diversity than did population DS (PPB = 65%, PPL = 72.73%). Because trees at DS are usually >100 years old, whereas those at DT and YKP are < 50 years old [19], range contraction within the past 50 years can be eliminated as a cause for this difference. Instead, patterns of gene flow, migration and population structure prior to range contraction can impact on modern populations [10], and may explain differences in genetic diversity between these *M. coriacea* populations. The fact that heterozygosity for SSR alleles was slightly higher in DS than the other two populations indicates that lower diversity here had not yet brought about inbreeding when the current generation was founded. However, the absence of seedlings from all three populations has prevented us from assessing whether range contraction will lead to an immediate inbreeding effect in the next generation.

It should be noted that there are some discrepancies between the results (e.g., levels of examined population’s heterozygosity, STRUCTURE and PCoA clusterings) from different markers, suggesting that the manner of polymorphism differs because of marker specificity. Contrasting patterns are commonly found when multiple markers are used to detect genetic structure, as for example in soybean [33], cashew [34], olive [35] and *Ficus carica* [36]. Such contrasts between markers could be due to any or all of the following causes: (1) these two marker systems sample genomic regions with different evolutionary modes; (2) ISSR is based on consensus markers, whereas the microsatellite markers used in this study were specifically developed for *M. coriacea*; and (3) ISSR and SSR have different modes of inheritance (*i.e.*, dominant *versus* codominant). In addition, some marker loci could be under selection, or tightly linked to loci under selection, meaning that a small minority of markers for either system might not be truly neutral, although neutrality of both ISSR and SSR markers is often assumed. To test directly for these possibilities is beyond the scope of the current study, but using two types of marker, and interpreting results of each with a degree of caution, can give a reasonable assessment of the true genetic situation for the examined populations. In the current study, population YKP was clearly separable from the other populations based on ISSR, but not SSR, markers. Hence, YKP might be the most distinct population, but SSR data indicates that this conclusion must be treated with caution.

### 3.3. Conservation

The maintenance of a maximal amount of genetic diversity is one of the major objectives for conserving endangered and threatened species [11], and the loss of genetic variation may largely limit adaptability to changing environments [37,38]. In the case of *M. coriacea*, AMOVA results shows that each of the three examined populations currently maintains a high level of within-population genetic diversity. Therefore, the endangered status of this species currently reflects a small number of extant individuals and very poor natural regeneration. Conservation measures should therefore focus on establishing large numbers of seedlings, both *in* and *ex situ*, involving many different individuals as parents, to preserve as much as possible of the existing genetic diversity in subsequent generations. Given the current lack of recruitment, establishing seedlings *in situ* will require management and co-operation from local communities.

In addition, the observed high diversity among relatively few adult trees also places a conservation value on each individual adult tree as a mini-reservoir of genetic diversity, in a way that would not apply for genetically depauperate, relict populations. Hence, conservation efforts to preserve each existing tree would also have a value in maintaining genetic diversity.

Conservation strategies may differ between sites. In DS, the oldest trees are protected by villagers as good luck symbols [19], so encouraging the locals to grow seedlings from these trees to bring luck to future generations might be effective. This is, however, the least diverse population. Individuals from DT and YKP occur in secondary vegetation and enjoy no protection of any kind [19]. Of these two populations, YKP might be the most distinct and hence of higher conservation value deduced from ISSR results.

Within each population, however, SSR data also indicated clusters of individuals more similar to one another than they were to others. For example, individuals DS1 and DS2 were genetically highly distinct from others is DS, and likewise YKP3, YKP4 and YKP6 were genetically distinct from others in YKP. Therefore, seedlings with a higher degree of heterozygosity could be generated by crossing individuals that, according to our results, are genetically dissimilar.

In the short term, conservation priorities must be to preserve as many as possible of the surviving individuals. In the medium term, new generations must be ensured, and in the long term, genetic health must be maintained. Therefore, *in-situ* efforts to conserve remaining habitats need to be combined with *ex-situ* research on seed propagation, with a view to establishing a new generation of plants both in cultivation and the wild. Should *in-situ* conservation be more successful at one site than others, then seedlings from all three sites could be established at the protected site.

## 4. Materials and Methods

### 4.1. The Study Species and Sampling Procedures

*Michelia coriacea* is diploid (2*n* = 38) [39]. The mature trees can reach up to 20–30 m in height and 50–60 cm in diameter [18,19,40], and live for at least 100 years. The species bears scented whitish or creamy yellow flowers with 6–7 tepals which are in anthesis from February to April [13,14].

The plant sampling and material collection were carried out in March and July 2007, and in August 2008. Prior to this, comprehensive field surveys had located only 15, 33 and 53 individuals at DS, DT and YKP, respectively [19]. It should be noted that seedling regeneration is not good, because no seedlings were detected in DS, and only a few seedlings were found in the other two populations. Therefore, we sampled all 15 mature individuals in DS, whereas 21 and 22 mature individuals were sampled from DT and YKP respectively (Figure 1 and Table 6). Healthy, young leaves were collected from each sampled individual, and dried in silica gel for subsequent DNA extraction.

### 4.2. DNA Extraction and PCR Amplification

Total DNA was extracted from the dried leaves following the modified CTAB method described by Doyle [41]. The purified total DNA was quantified by gel electrophoresis and its quality verified by spectrophotometry. DNA samples were stored at −20 °C.

One hundred ISSR primers from the University of British Columbia (UBC) were initially screened on eight randomly selected individuals for PCR. Of these, ten primers which consistently amplified well, and produced polymorphic bands, were selected for analyzing (Table 1). To ensure repeatability of amplification and scoring, all loci were amplified 2 times independently and run on separate gels. PCR reactions were performed in a reaction volume of 15 μL, containing 9.92 μL ddH_2_O, 0.4 μL formamide, 1.5 μL 10× PCR buffer (Mg^2+^ Plus), 0.25 mM of each dNTP, 0.9 U Taq polymerase, 0.6 μM primers, 0.6 μL template DNA (approximate 50 ng). Cycling conditions consisted of an initial denaturation step at 97 °C for 4 min, followed by 35 cycles of denaturation at 94 °C for 1 min, annealing temperature for 1 min (Table 1), extension at 72 °C for 1.5 min, and finally 10 min at 72 °C for final extension. Amplification products were detected by using 1.6% agarose gel stained with 0.5 pg/mL ethidium bromide, and were electrophoresed in 1× Tris-borate-EDTA (TBE) buffer (pH 8.0) at 85 V for 2 h. The bands were visualized under UV light.

SSR genotyping was performed according to the methods described in Zhao *et al.* [40] at loci MC2, MC3, MC8, MC34, MC35, MC41, MC45, MC48, MC49, MC64, and MC66 (Table 2). Briefly, SSR loci were amplified by PCR in a final volume of 15 μL containing 7.5 μL 2× Taq PCR MasterMix (Tiangen; 0.1 U Taq Polymerase/μL, 0.5 mM dNTP each, 20 mM Tris-HCl, 100 mM KCl, 3 mM MgCl_2_), 20–50 ng genomic DNA. The amplification profiles included initial denaturation at 94 °C for 3 min; followed by 35–40 cycles of 30 s at 94 °C, 30 s at 54–68 °C and 1 min at 72 °C (Table 2); then final extension at 72 °C for 7 min. The amplified product was then separated on 6% denaturing polyacrylamide sequencing gels using silver staining. A 20 bp DNA ladder standard (Fermentas) was used as standard for scoring.

### 4.3. Data Analysis

#### 4.3.1. ISSR Data

Amplified ISSR fragments showing consistent amplification were scored manually as present (1) or absent (0) for each sample to form a binary matrix. The POPGENE program Version 1.31 [42] was employed, assuming Hardy-Weinberg equilibrium, to obtain the genetic diversity parameters within each population: percentage of polymorphic bands (PPB), private bands (referring to bands found only within one population), observed allele number per locus (*A*_o_), effective allele number per locus (*A*_e_), Nei’s gene diversity (*H*) and Shannon index of diversity (H_pop_) [31]. Genetic diversity parameters were also calculated both at species and population levels.

Genetic differentiation among populations was analyzed using Nei’s gene diversity statistics [27]. The gene flow was estimated by using the equation *N*_m_ = (1 − *G*_st_)/4*G*_st_, where *N*_m_ is the number of migrants per generation [43]. To infer population structure and assign individuals to populations, the program STRUCTURE version 2.3.1 was used [44], following the methods described by Ma *et al*. [45]. We adopted the admixture model with correlated allele frequencies. No prior knowledge of the species was included in the analyzed data set. To determine the optimal number of groups (*K*), we ran STRUCTURE with *K* varying from 1 to 10, with five runs for each *K* value. Previous studies have found that, in many cases, the posterior probability for a given *K* increases slightly, even after the real *K* is reached [46]. Therefore, we used an *ad hoc* statistic, Δ*K*, to determine the true value of *K* [47]. Our parameters were 100,000 burn-in periods and 10,000 MCMC repetitions after burn-in. Furthermore, principal co-ordinate analysis (PCoA) in GenAlEx 6.0 was employed to examine further the genetic relationships among detected populations on the basis of the same ISSR data [48].

The AMOVA (analysis of molecular variance) was performed through Arlequin 3.0 program [49] to describe variance components and their significance levels for variation among individuals within and among the populations.

#### 4.3.2. SSR Data

To estimate overall genetic diversity, the following measures were calculated for each SSR primer locus and each population using POPGENE program Version 1.31 [42]: observed allele number per locus (*A*_o_), effective allele number per locus (*A*_e_), observed heterozygosity (*H*_o_), expected heterozygosity (*H*_e_), and the number of private loci, *i.e.*, those found only in a single population. The same program was used to test for deviations from Hardy-Weinberg equilibrium (HWE) and pair-wise linkage disequilibrium (LD). To measure the marker polymorphism, the polymorphism information content (PIC) for each SSR Marker was calculated according to the formula PIC = 1 − ∑*P_i_* ^2^, where *P_i_* is the frequency of the allele for each SSR marker locus [50].

Wright’s *F*_st_ was estimated by a weighted analysis of variance with the GENEPOP 4.0 program [51]. In addition, the amount of gene flow (*N*_m_) among populations was estimated by an indirect genetic differentiation method based on *F*_st_ value, *N*_m_ = (1 − *F*_st_)/4*F*_st_. Partitioning of genetic variation within and among populations was further performed by analysis of molecular variance using the Arlequin 3.0 program [49]. For these SSR data, genetic relationships among three populations were also examined using STRUCTURE and GenAlEx 6.0 with the same methods described as ISSR markers above.

## 5. Conclusions

In summary, our results from both ISSR and SSR markers show that a relatively high level of genetic diversity and low levels of genetic differentiation among populations exist in the critically endangered plant *Michelia coriacea*. Furthermore, Bayesian model-based STRUCTURE and PCoA analysis could not reveal a clear separation between populations, although YKP was differentiated from the other two populations by ISSR markers. We therefore presume that the fragmented habitat and the isolated populations of *M. coriacea* may be due to very recent contraction of its range. Based on this data, conservation strategies for this critically endangered species were also proposed.

## Figures and Tables

**Table 1:** ISSR primers used for DNA amplifications, PCR annealing temperature (*T*_a_), number of bands per primer, number and percentage of polymorphic bands.

Primer code	Primer sequence (5′–3′)	*T*_a_ (°C)	No. of bands per primer	No. of polymorphic bands	Polymorphism (%)
17	(AG)_8_TC	52	10	10	100.00
815	(CT)_8_G	52	13	12	92.31
824	(TC)_8_G	50	8	7	87.50
834	(AG)_8_YT	52	9	9	100.00
836	(AT)_8_YA	50	7	7	100.00
840	(GA)_8_YT	52	15	15	100.00
846	(CA)_8_RT	52	8	8	100.00
848	(CA)_8_RG	52	15	15	100.00
866	(CTC)_5_	52	13	13	100.00
895	AGA GTT GGT AGC TCT TGA TC	52	12	10	83.33
Total			110	106	96.36

Y = (C, T); R = (A, G).

**Table 2:** Genetic diversity within the *Michelia coriacea* populations as indicated by ISSR markers.

Population	Polymorphic bands			*A*_o_	*A*_e_	*H*	*H*_pop_

	No.	%	PrB				
DS	66	60	0	1.600 (0.492)	1.309 (0.349)	0.188 (0.189)	0.286 (0.271)
DT	90	81.82	9	1.818 (0.388)	1.386 (0.352)	0.234 (0.179)	0.363 (0.245)
YKP	84	76.36	8	1.764 (0.427)	1.430 (0.357)	0.256 (0.184)	0.387 (0.258)
Average	80	72.73	5.67	1.727 (0.436)	1.375 (0.353)	0.226 (0.184)	0.345 (0.258)
Species level	106	96.36		1.964 (0.188)	1.460 (0.315)	0.283 (0.156)	0.436 (0.204)

PrB: private bands; *A*_o_: observed allele number per locus; *A*_e_: effective allele number per locus; *H*: Nei’s gene diversity; *H*_pop_: Shannon’s information index; Values in brackets are standard deviations.

**Table 3:** Analyses of molecular variance (AMOVA) for *Michelia coriacea* by ISSR and SSR markers.

Source of variation	d.f.	Sum of squares	Variance component	Percentage of variance	*p* value
**ISSRs**					
Among population	2	165.176	3.676	22.839	<0.01
Within population	55	683.134	12.421	77.161	<0.01

**SSRs**					
Among population	2	46.160	0.913	13.900	<0.01
Within population	55	311.099	5.656	86.100	<0.01

**Table 4:** SSR primers used for DNA amplifications, locus, repeat motif, primer size (bp), PCR annealing temperature (*T*_a_), number of alleles (*A*), observed heterozygosity (*H*_o_), excepted heterozygosity (*H*_e_) and polymorphism information content (PIC).

Locus	Repeat motif	Size range (bp)	*T*_a_ (°C)	*A*	*H*_o_	*H*_e_	PIC
MC2	(CA)_8_	192–206	60	3	0.535	0.582	0.517
MC3	(CT)_16_	292–306	68	3	0.500	0.624	0.553
MC8	(TC)_15_	189–211	58	6	0.759	0.782	0.750
MC34	(TC)_4_T_3_(TC)_3_-(TC)_4_	156–160	62	4	0.621	0.638	0.584
MC35	(CT)_3_-(CT)_4_-(CT)_3_	375–381	62	3	0.276	0.439	0.489
MC41	(TTTC)_4_-(CT)_10_	216–232	60	3	0.603	0.497	0.440
MC45	(CT)_10_T_2_(CT)_2_	116–124	62	3	0.172	0.247	0.433
MC48	(TC)_8_-(TC)_5_-(TC)_6_	137–157	58	5	0.328	0.496	0.417
MC49	(TC)_8_	140–158	64	7	0.224	0.531	0.480
MC64	(CT)_4_-(CT)_4_-(CT)_2_	248–252	60	2	0.259	0.274	0.436
MC66	(CT)_8_	295–305	54	6	0.257	0.449	0.421
Mean				4.091	0.412	0.505	0.502
St. Dev				1.640	0.197	0.157	

**Table 5:** Genetic diversity within the *Michelia coriacea* populations by SSR markers.

Population	PPL (%)	PrA	*A*_o_	*A*_e_	*H*_o_	*H*_e_
DS	72.73	2	3.455 (1.214)	2.170 (0.630)	0.533 (0.257)	0.498 (0.168)
DT	91.90	3	3.455 (1.036)	1.946 (0.604)	0.335 (0.207)	0.429 (0.210)
YKP	81.82	2	3.455 (1.036)	2.236 (0.962)	0.407 (0.234)	0.485 (0.190)
Average	82.15	2.33	3.455 (1.095)	2.117 (0.732)	0.425 (0.232)	0.470 (0.189)

PPL: percentage of polymorphic loci; PrA: private allele for population; *A*_o_: observed allele number per locus; *A*_e_: effective allele number per locus; *H*_e_: expected heterozygosity; *H*_o_: observed heterozygosity; Values in brackets are standard deviations.

**Table 6:** Known populations of *Michelia coriacea* examined for ISSR and SSR analyses with population code, locality, altitude, location coordinates, numbers of individuals sampled, sample code and voucher numbers.

Code	Locality	Altitude (m)	Latitude (N)	Longitude (E)	*N* (sample code)	Voucher number
DS	Daping-Shipen, Malipo, Yunnan, China	1504–1600	23°5′–23°7′	104°35′–104°40′	15 (DS1-DS15)	Z-005–007, Z-036–042, Z-051–053.
DT	Dongma-Tiechang, Xicou and Malipo, Yunnan, China	1413–1496	23°2′–23°25′	104°54′–104°57′	21 (DT1-DT21)	Z-003–004, Z-009–25, Z-049.
YKP	Yang-Kai-Ping, Malipo, Yunnan, China	1289–1305	23°7′–23°8′	104°51′	22 (YKP1-YKP22)	Z-YKP-01–22.

All vouchers are deposited at KUN (Herbarium of Kunming Institute of Botany, CAS).
